# The Nutritional and Antioxidant Potential of Artisanal and Industrial Apple Vinegars and Their Ability to Inhibit Key Enzymes Related to Type 2 Diabetes In Vitro

**DOI:** 10.3390/molecules27020567

**Published:** 2022-01-17

**Authors:** Driss Ousaaid, Hassan Laaroussi, Hamza Mechchate, Meryem Bakour, Asmae El Ghouizi, Ramzi A. Mothana, Omar Noman, Imane Es-safi, Badiaa Lyoussi, Ilham El Arabi

**Affiliations:** 1 Laboratory of Natural Substances, Pharmacology, Environment, Modeling, Health and Quality of Life (SNAMOPEQ), Department of Biology, Faculty of Sciences Dhar El Mahraz, University Sidi Mohamed Ben Abdellah, Fez 30000, Morocco; driss.ousaaid@usmba.ac.ma (D.O.); hassan.laaroussi@usmba.ac.ma (H.L.); meryem.bakour@usmba.ac.ma (M.B.); asmae.elghouizi@usmba.ac.ma (A.E.G.); lyoussi@gmail.com (B.L.); ilham.elarabi@usmba.ac.ma (I.E.A.); 2 Laboratory of Inorganic Chemistry, Department of Chemistry, University of Helsinki, P.O. Box 55, FI-00014 Helsinki, Finland; Imane.essafi1@usmba.ac.ma; 3 Department of Pharmacognosy, College of Pharmacy, King Saud University, Riyadh 11451, Saudi Arabia; rmothana@ksu.edu.sa (R.A.M.); onoman20@gmail.com (O.N.)

**Keywords:** nutrition, antioxidant activity, artisanal apple vinegar, industrial apple vinegar, diabetes, α-amylase, α-glucosidase

## Abstract

The main objective of the current study was to determine the physicochemical properties, antioxidant activities, and α-glucosidase and α-amylase inhibition of apple vinegar produced by artisanal and industrial methods. Apple vinegar samples were analyzed to identify their electrical conductivity, pH, titratable acidity, total dry matter, Brix, density, mineral elements, polyphenols, flavonoids, and vitamin C. The antioxidant activity of apple vinegar samples was evaluated using two tests, total antioxidant capacity (TAC) and DPPH radical scavenging activity. Finally, we determined α-glucosidase and α-amylase inhibitory activities of artisanal and industrial apple vinegar. The results showed the following values: pH (3.69–3.19); electrical conductivity (2.81–2.79 mS/cm); titratable acidity (3.6–5.4); ash (4.61–2.90); °Brix (6.37–5.2); density (1.02476–1.02012), respectively, for artisanal apple vinegar and industrial apple vinegar. Concerning mineral elements, potassium was the most predominant element followed by sodium, magnesium, and calcium. Concerning bioactive compounds (polyphenols, flavonoids, and vitamin C), the apple vinegar produced by the artisanal method was the richest sample in terms of bioactive compounds and had the highest α-glucosidase and α-amylase inhibition. The findings of this study showed that the quality and biological activities of artisanal apple vinegar were more important than industrial apple vinegar.

## 1. Introduction

In Morocco, *Malus domestica* is the tree of mountainous areas, and it has constantly been the keystone of the agricultural activity of the Amazigh people of the mountains such as Midelt, Imilchil, Azrou, and Emmouzzer [1]. An apple is a very perishable fruit, and due to its functional properties, it is used as a raw material in the production of numerous by-products with high value added, such as vinegar [2]. The commercial exploitation of vinegar is linked to its composition, which includes high levels of bioactive substances, vitamins, and mineral elements [3].

Recently, by-products have been increasing in popularity due to their functional properties [4]. This growing demand has occurred with studies of the beneficial effects of these natural products [5]. In Morocco, a wide range of local products including fermented products such as olive and apple have a variety of culinary and medicinal uses [6,7]. Therefore, several traditional food products are still consumed and appreciated by consumers, such as apple vinegar, which constitutes a base of many food industries. In the last decade, the food challenge is no longer just quantitative but also qualitative.

Apple vinegar production involves sequential ethanolic and acetic fermentation of apple. The first step requires the absence of oxygen, while the second step requires its presence [8]. Apple-vinegar making was performed by several methods including the slow method or artisanal method and the speed method via submerged culture [8,9]. The traditional process takes as long as a few months, and leads to a higher quality product [10,11]. Additionally, the longer period contributes to the quality of apple vinegar because it leads to the biological interaction between microorganisms and wood barrels, which generates new compounds with biological functions. Otherwise, the submerged culture method requires a high oxygen supply to accelerate the apple vinegar-making process. Despite the short duration of apple vinegar-making, the excess of oxygen accentuates the degradation of bioactive molecules and does not allow the production of newly generated biologically active compounds like the aforementioned method [11].

The production of artisanal apple vinegar requires minimal technological interventions using the indigenous microorganisms that occur naturally in apple fruits. Yeast microflora ferments the apple sugars in the absence of oxygen. After a few months, the barrels used to prepare apple vinegar allow oxygen to enter, and then the alcohol produced during the first step is converted naturally to acetic acid by indigenous acetic bacteria. The resultant vinegar was filtrated and packaged then sold in different cooperative markets [12]. 

**Figure 1:**
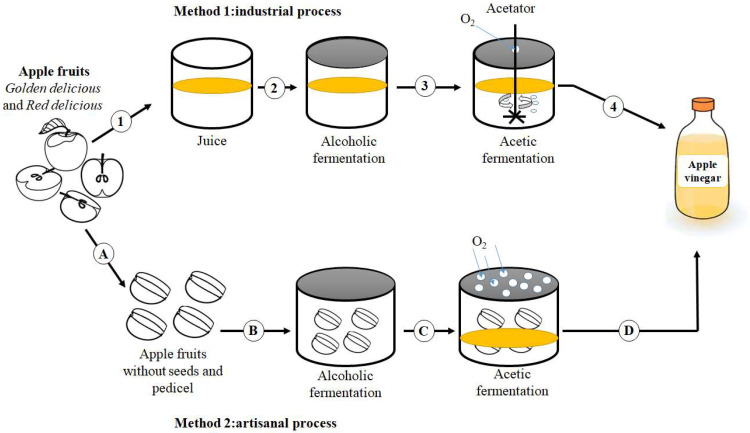
Industrial and artisanal procedures to produce apple vinegar. 1: apple juice extraction; 2: alcoholic fermentation; 3: acetic fermentation; 4: production of vinegar via the industrial method; A: usage of apple fruits without seeds and pedicel; B: alcoholic fermentation; C: acetic fermentation; D: production of vinegar via the artisanal method.

The industrial method (submerged culture) requires technological intervention that confers the proper control of fermentation conditions. Industrial apple vinegar is made by fermenting apple juice. The first step consists of inoculating the microbial culture (*Saccharomyces cerevisiae*) into apple juice for alcohol production through anaerobic fermentation, while the second step requires the incorporation of oxygen using a high-speed motor that breaks down the air that is brought down from a stainless steel tank into tiny bubbles and is forced into the solution of alcoholic liquid. The presence of oxygen leads acetic bacteria to transform alcohol into acetic acid (Figure 1) [12]. 

Vinegar has been broadly used in folk remedies to treat different diseases [13,14]. Among ancient prescriptions described in the Chinese Book Fifty-Two diseases (300 BC) [13]. Multiple studies have reported the beneficial effects of apple vinegar such as anti-inflammatory, antitumor, antidiabetic, and antihypertensive benefits [11,13,14]. Several reports mentioned that the intake of vinegar helped in controlling blood glucose levels and that it has an inhibitory effect on α-glucosidase [15,16].

The inhibition of digestive enzymes constitutes a key factor to reduce the postprandial response after meals intake. Based on this rationale, our study provides the first intervention to assess the vinegar quality criteria such as physicochemical properties and antioxidant activities of vinegar and to determine the ability of artisanal and industrial apple vinegar to inhibit α-glucosidase and α-amylase enzymes.

## 2. Results and Discussion

### 2.1. Physicochemical Parameters

The physicochemical parameters of the vinegar samples are summarized in Table 1. The pH values ranged between 3.19 ± 0.02 (industrial apple vinegar) and 3.69 ± 0.00 (artisanal apple vinegar). The results are in line with data reported by previous studies [17,18,19]. The difference in pH values may be due to the microorganism’s development and the control of the aeration system in the industrial process. The electrical conductivity values ranged between 3.60 ± 0.21 and 5.20 ± 0.14 mS/cm for artisanal apple vinegar and industrial apple vinegar, respectively. These results are lower than those documented by Siddeeg et al. [19]. The Brix index values oscillate between a minimum recorded at the industrial apple vinegar (5.20 ± 0.14) and a maximum at the artisanal apple vinegar (6.37 ± 0.04). Results obtained in this study are in accordance with the data reported by Siddeeg et al. [19], and higher than those documented by Sengun et al. [17], while the density of vinegar was similar for industrial and artisanal vinegar, (1.02 ± 0.00). Siddeeg et al. stated that the density of vinegar was higher than this obtained from our study [19].

### 2.2. Mineral Contents

Table 2 shows the average content of K, Na, Ca, Mg, P, Fe, Zn, Mn, Cu, Pb, Cr, and Cd elements that were determined by the ICP atomic absorption method. The results showed that the two vinegar samples are very rich in these mineral elements. Potassium was the most predominant element in both samples of vinegar. The mineral composition of the studied vinegar samples is closely related to the composition of the fruit of each variety and is influenced by the geographical, edaphic origin, and the fertilizers used which have a direct impact on the mineral composition of the harvested fruits [20]. The use of heavy metals such as heavy metal lead (Pb) and cadmium (Cd) is limited in the food industry [21]. The obtained results are in agreement with the recommendation of the Codex Alimentarius Commission (CODEX), which set the maximum content of Pb at 0.2 mg/L [22]. Interestingly, heavy metals (Pb, Cr, and Cd) were not detected in both vinegar samples studied.

### 2.3. Bioactive Molecules and Antioxidant Activity

The bioactive compounds and antioxidant activity are summarized in Table 3. The highest values of total phenolic content (TPC), total flavonoid content (TFC), and vitamin C were registered in the artisanal apple vinegar, while the lowest values were found in industrial apple vinegar. Concerning the antioxidant activity of vinegar samples evaluated (TAC and DPPH), the artisanal vinegar has the higher total antioxidant capacity (9.17 ± 0.86 mg AAE/100 mL) and the lowest value was registered in industrial apple vinegar (4.22 ± 0.26 mg AAE/100 mL). Concerning the ability of vinegar to act as hydrogen donors, the results indicated that the artisanal vinegar showed the highest potent DPPH radical scavenger capacity (0.31 ± 0.01 µL/mL), while industrial vinegar has the lowest capacity (0.90 ± 0.03 µL/mL). Both vinegar samples exhibited high antioxidant ability towered DPPH free radical as compared to Trolox (IC_50_ = 10.81 ± 0.10 µg/mL). The results of the phenolic compounds analysis revealed that arbutin (32.60%), apigenin (16.48%), and Transferulic acid (11.94%) were the major compounds found in AAV, while arbutin (45.59%), trans-cinnamic acid (4.99%), and P-coumaric acid (4.56%) were the major compounds found in IAV. Published studies on the antioxidants activity of apple vinegar are reported that radical scavenging activity of industrial apple vinegar from China was 0.0178 mmol TEAC/mL for artisanal apple vinegar [23] and 0.21 mg TE/mL for industrial apple vinegar from Turkey [24]. 

The bioactive properties of vinegar are influenced by multiple factors such as raw material, the technique of production, and geographical conditions. Previous studies reported that apple vinegar exhibited a remarkable antioxidant activity [24,25,26]. It has been proved that apple vinegar has several pharmacological properties including antidiabetic effect, antibacterial effect, antihypertensive effect, and anti-inflammatory effect [25,27,28,29,30,31,32].

### 2.4. α-Amylase and α-Glucosidase

The management of metabolic disorders requires the inhibition of carbohydrates hydrolyzing enzymes as the first line of treatment [33]. The inhibition of carbohydrate hydrolyzing enzymes could decrease postprandial hyperglycemia [34]. Numerous studies proved the ability of apple vinegar to reduce hyperglycemia [35,36,37,38]. In this context, the activity on α-glucosidase and α-amylase inhibition of the two samples of apple vinegar produced by different techniques were evaluated (Table 4). The obtained results revealed that the AAV had the highest level of activity against α-glucosidase with IC_50_ = 156.53 ± 0.07 µg/mL and α-amylase with IC_50_ = 16.32 ± 0.01 µg/mL, as compared to the IAV (IC_50_ = 152.86 ± 0.06 µg/mL for α-amylase and IC_50_ = 4024.28 ± 5.12 µg/mL for α-glucosidase. These values were lower than those expressed by acarbose, in which the IC_50_ values were 35.42 ± 1.00 µg/mL and 11,000 ± 1.00 µg/mL for α-amylase and α-glucosidase, respectively. The inhibition of both enzymes contributes to the management of type 2 diabetes through suppression or interrupting glucose generation carbohydrates absorption [33]. Diminution of carbohydrates hydrolyzing is considered as one of the successful approaches to reduce hyperglycemia and hyperlipidemia [38]. There are many bioactive substances such as terpenes (22-a hydroxychiisanoside) and alkaloids (palmatine) that can inhibit α- glucosidase and α-amylase enzymes [39,40]. However, bioactive molecules which can inhibit the alpha-glucosidase and alpha-amylase enzymes and at the same time which possess remarkable antioxidant activities could be very good candidates for controlling blood sugar levels and the oxidative stress that accompanies the diabetes. For this reason, in the present study, we have chosen to focus on the analysis and the identification of antioxidant compounds in vinegar [41]. 

The phytochemical quantification revealed the presence of phenolic compounds which exhibit a good enzyme inhibition activity [42]. It has been shown that flavonoids have a higher inhibition activity [42]. Individual phenolic compounds present in vinegar including gallic acid, chlorogenic acid, ferulic acid, caffeic acid, and catechin showed a significant inhibition through binding carbohydrate hydrolyzing enzymes [42]. In addition to phenolic acids, arbutin, glycosylated hydroquinone presented as a predominant bioactive molecule in AAV (32.60%) and IAV (45.58%) has been documented to have in vivo and in vitro antihyperglycemic activity [43,44]. Yousefi and coworkers have reported that arbutin displayed potent α-amylase and α-glucosidase inhibitory action [43]. In addition, apigenin was recognized as a mono-therapeutic agent, inhibiting both α-glucosidase and α-amylase enzymes [45]. UHPLC analysis showed that AAV contains a high amount of apigenin (16.48%) as compared to the IAV (2.53%) which might explain its powerful inhibitory action against these two digestive enzymes. 

Moreover, syringic, vanillic, and sinapic acids, present in both artisanal and industrial vinegars (Table 5), inhibit α-amylase and α-glucosidase activities through a specific binding between methoxy group of their aromatic ring and the active sites of these enzymes, which could be the target pathway involved by other hydroxycinnamic and hydroxybenzoic acids identified in our vinegar samples [45].

Furthermore, acetic acid, as a major organic acid present in apple vinegar, was suggested as a key factor in reducing disaccharidase activity, intestine maltase, lactase, and sucrase activities [38,46]. In the same context, the administration of acetic acid suppressed carbohydrates hydrolyzing enzymes (sucrase, maltase, lactase, and trehalase) [46]. Apple vinegar consumption decreases pH, which could inactivate α-amylase in different stages of digestion through the digestive tube [47]. Digestive enzymes’ inhibitory effects of organic acids present in vinegar are very interesting. It has been previously reported that the acid citric was the most active organic acid compared to other acids (malic acid, tartaric acid, and succinic acid) [48]. Interestingly, apple vinegar contains a cocktail of bioactive compounds including phenolic components. It has been documented that phenolic compounds exhibited a stronger inhibition against α-glucosidase and α-amylase [49,50,51]. Finally, the synergic effect of different bioactive compounds of apple vinegar gives this bioproduct its ability to control the first line of management of diabetes by reducing the availability of monosaccharides. 

## 3. Materials and Methods

### 3.1. Vinegar Preparation

Apple vinegar samples were produced and sold in the Midelt area (32°41′06.7″ N 4° 44′42.4″ W). The samples were prepared locally by cooperatives based on two techniques (artisanal and industrial methods). The production of apple vinegar involves sequential ethanolic and acetic fermentation.

### 3.2. Physicochemical Properties Evaluation

The purchased apple vinegar samples were filtered and then the filtrates were used to evaluate the following parameters: pH was determined using a pH meter, and electrical conductivity was measured using an electrical conductivity cell. The titratable acidity was determined by titrating the apple vinegar with 0.1 M sodium hydroxide solution. The total soluble solids and the density were measured using a refractometer [18], while the mineral elements were determined by the calcination method using ICP-AES as described previously by Laaroussi et al. [52]. 

### 3.3. Bioactive Substances

#### 3.3.1. Total Phenolic Content

The quantification of the polyphenol compounds was determined using the Folin-Ciocalteau method as described previously by Laaroussi et al. [52]. The result was expressed as mg Gallic acid equivalent per 100 mL of vinegar (mg GAE/100 mL).

#### 3.3.2. Total Flavonoid Content

The total flavonoid content of vinegar samples was carried out according to the method of Kong et al. [53], with some modifications. Briefly, 0.1 mL of vinegar was mixed with 150 µL of sodium nitrite (5%), 150 µL of Alcl_3_ solution (10%), and 200 µL of sodium acetate. After one hour of incubation, the absorbance of the mixture was measured at 510 nm. Then, the values of TFC were expressed as mg quercetin equivalent per 100 mL of the vinegar (mg QE/100 mL). 

#### 3.3.3. Ascorbic Acid

The quantification of ascorbic acid was carried out by titration with iodine solution (5 g of potassium iodide (KI), 0.268 g of potassium iodate (KIO_3_), and 30 mL of 3 M sulfuric acid were mixed in 500 mL of distilled water) and using starch 1% (*w*/*v*) as indicator solution [54]. The concentration of ascorbic acid was calculated as mg per 100 mL of vinegar.

### 3.4. Antioxidant Activity

The evaluation of the antioxidant activity of the studied vinegar samples was carried out using two techniques. The total antioxidant capacity (TAC) was determined using the phosphomolybdenum method as described previously by Laaroussi et al. [18]. The total antioxidant capacity of studied samples was expressed in equivalent ascorbic acid per 100 mL of vinegar (mg AAE/100 mL of vinegar).

The free radical scavenging ability of our samples (DPPH) was determined according to the method described previously by Laaroussi et al. [18]. The antioxidant activity was estimated based on the percentage of inhibition using the following formula:

(1) $$I\;\left(\%\right)=\left(A_{0}-\frac{A_{1}}{A_{0}}\right)\times 100$$

*A*_0_ is the absorbance of the control; *A*_1_ is the absorbance of the sample.

The values providing 50% free radical scavenging (IC_50_) were deducted from the graph inhibition percentage. Results were expressed as µL/mL.

### 3.5. Identification and Quantification of Phenolic Compounds of Apple Vinegar Samples

Aliquots of apple cider vinegar (80 mg) were treated with 1 mL of ethanol. The Eppendorf was vortexed and incubated in a sonicator bath at 45 °C for 60 min. Qualitative analysis was carried out resorting to a Shimadzu Ultra-High-Performance. Liquid Chromatograph (Nexera XR LC 40), which was combined with an MS/MS detector (LCMS 8060, Shimadzu Italy, Milan, Italy). The MS/MS was wielded with electrospray ionization and controlled by Lab Solution software, which provided at once a fast transition from a low-energy scan 4 V (full scan MS) to a high-energy scan (10–60 V ramping) during a single LC run. The source parameters were fixed as follows: nebulizing gas flow: 2.9 L/min, heating gas flow: 10 L/min, interface temperature: 300 °C, DL temperature: 250 °C, heat block temperature: 400 °C, and drying gas flow: 10 L/min. Flow injection was used to perform the analysis (meaning that there was no chromatographic separation), with the mobile phase that consist of acetonitrile: water + 0.01% formic acid (5:95, *v*/*v*). The instrument was set for a SIM experiment in negative mode (only syringic acid in positive ESI). The identification of the molecules was confirmed by comparing the typical fragment identified with those in our in-house developed library of molecules and a molecule was considered “positive” if its area under the curve was higher in magnitude than those of the blank and, accordingly, the abundance of the molecules was estimated (See Supplemental Materials). Differentiation between closely similar structures was performed by “time of flight”, as the instrument was set to acquire the molecular weight in the third quadrupole.

### 3.6. α-Amylase Inhibition Assay 

The method described previously by Ferreira-Santos et al. was selected to determine α-amylase inhibitory activity [55]. In brief, 500 µL of enzyme and apple vinegar were mixed and incubated for 15 min at 37 °C. Then, starch solution (1%) was prepared and 500 µL was added to the mixture and incubated at 37 °C for 15 min. Furthermore, 1 mL of dinitrosalicylic acid was added to the mixture reagent and incubated in a boiling water bath. The absorbance was read at 540 nm after dilution 10 times the final mixture. Acarbose and ethanol 70% were used as a positive and negative control, respectively. 

### 3.7. α-Glucosidase Inhibition Assay

A mixture of different concentrations of apple vinegar and p-nitrophenyl-R-d-glucopyranoside (pNPG, 3 mM) was mixed with α-glucosidase solution (10 UI/mL). The mixture obtained was incubated at 37 °C for 15 min. Na_2_CO_3_ solution (1 M) was used to stop the reaction. Finally, the absorbance was read at 400 nm [55].

α-amylase and α-glucosidase inhibitory activity (%) was calculated using Equation (1). 

The values providing 50% of the activity of α-amylase and α-glucosidase (IC_50_) were calculated from a dose–response curve, and the obtained results were expressed as µg/mL.

### 3.8. Statistical Analysis 

The analyses of apple vinegar were carried out in triplicate and the results were presented as mean ± SD. Statistical analysis was carried out by GraphPad Prism software (version 6.0; GraphPad Software, Inc., San Diego, CA, USA). Student *t*-test was used to compare the two apple vinegar samples, *p* < 0.05 was considered significant. 

## 4. Conclusions

In this study, the comparison between artisanal apple vinegar and industrial apple vinegar showed that artisanal apple vinegar has a higher quality with regard to physicochemical properties and bioactive molecules contents. Industrial vinegar is distinguished by its speed of manufacturing; however, with this speed, it loses some bioactive compounds and minerals. The results have demonstrated that artisanal vinegar is more effective in inhibiting α-glucosidase and α-amylase enzymes as compared to industrial apple vinegar. Therefore, we can conclude that using the artisanal method when making vinegar is the most appropriate method to ensure good quality and to produce healthy products. 

## Figures and Tables

**Table 1:** Physicochemical parameters of vinegar samples.

Samples	pH	Electrical Conductivity	Titratable Acidity	°Brix	Density
AAV	3.69 ± 0.00 ***	2.81 ± 0.01	3.60 ± 0.21	6.37 ± 0.04 **	1.02 ± 0.00
IAV	3.19 ± 0.02	2.79 ± 0.00	5.40 ± 0.14 **	5.20 ± 0.14	1.02 ± 0.00

AAV: Artisanal apple vinegar, IAV: Industrial apple vinegar, data are expressed as mean ± SD. ** *p* < 0.01 and *** *p* < 0.001.

**Table 2:** Content of mineral elements of vinegar samples.

Samples	K mg/L	Na mg/L	Ca mg/L	Mg mg/L	P mg/L	Fe mg/L	Zn mg/L	Mn mg/L	Cu mg/L	Pb mg/L	Cr mg/L	Cd mg/L
AAV	39.15 ± 0.07 ***	6.12 ± 0.10 ***	4.05 ± 0.06 ***	5.33 ± 0.11 ***	1.33 ± 0.02	0.032 ± 0.01 **	1.34 ± 0.06 ***	ND	ND	ND	ND	ND
IAV	32.90 ± 0.05	3.44 ± 0.01	2.63 ± 0.07	3.71 ± 0.17	1.36 ± 0.03 **	0.023 ± 0.01	0.24 ± 0.02	ND	ND	ND	ND	ND

AAV: Artisanal apple vinegar, IAV: Industrial apple vinegar, ND: not detected, data are expressed as mean ± SD. ** *p* < 0.01 and *** *p* < 0.001.

**Table 3:** Bioactive compounds and antioxidant activities of industrial and artisanal vinegars.

Samples	TPC mgGAE/100 mL	TFC mgQE/100 mL	Vit C mg/100 mL	TAC mg AAE/100 mL	DPPH IC_50_ (µL/mL)
AAV	106.91 ± 1.64 ***	11.36 ± 0.06 ***	15.4 ± 0.01 ***	9.17 ± 0.86 ***	0.31 ± 0.01
IAV	68.08 ± 0.23	3.47 ± 0.25	13.64 ± 0.01	4.22 ± 0.26	0.90 ± 0.03 ***
Trolox	-	-	-	-	10.81 ± 0.10

AAV: Artisanal apple vinegar, IAV: Industrial apple vinegar, TPC: total phenolic content TFC: total flavonoid content CT: GAE: gallic acid equivalent QE: quercetin equivalent, Vit C: Vitamin C, TAC: Total antioxidant capacity, DPPH: 1,1-diphenyl-2-picrylhydrazyl-2,2-diphenyl-1-picrylhydrazyl, data are expressed as mean ± SD. *** *p* < 0.001.

**Table 4:** α-glucosidase and α-amylase inhibitory activities of artisanal and industrial apple vinegar.

Samples	α-Amylase IC_50_ (µg/mL)	α-Glucosidase IC_50_ (µg/mL)
AAV	16.32 ± 0.01	156.53 ± 0.07 ***
IAV	152.86 ± 0.06	4024.28 ± 5.12 ***
Acarbose	35.42 ± 1.00	1100 ± 1.00

Data are expressed as mean ± SD. *** *p* < 0.001.

**Table 5:** Phenolic compounds of apple vinegar samples.

Phenolic Compounds	Concentration (%)	
	AAV	IAV
Quercetin	1.38	ND
P-coumaric	2.30	4.56
Oleochantal	1.15	0.91
Hydroxytyrosol	0.18	ND
Transferulic acid	11.94	4.32
Oleuropein	0.48	0.85
Hesperetin	0.55	0.40
Trimethoxyflavone	0.64	0.71
Arbutin	32.60	45.59
Rosmarinic acid	1.38	3.14
Ursolic acid	0.90	0.88
Apigenin	16.48	2.54
Amentoflavone	0.45	0.70
Luteolin	1.54	1.52
Quercetin-3-*O*-glucoside	0.64	0.58
Quercetin-3-*O*-glucuronic acid	0.81	0.85
Kaempferol-3-*O*-glucose	ND	0.48
Quercetin-3-*O*-hexose deoxyhexose	0.05	0.52
Isorhamnetin-3-*O*-rutinoside	0.04	0.62
Isorhamnetin-7-*O*-Pentose/luteoilin 7-*O*-glucoside	0.63	1.89
Kaempferol-3-*O*-glucuronic acid	0.80	ND
Kaempferol-3-*O*-pentose	ND	0.76
Kaempferol-3-*O*-hexose deohyhexose	ND	0.35
Protocathecoic acid	0.12	0.38
Vanillic acid	0.39	0.68
Syringic acid	4.07	3.22
P-hydroxybenzoic\salicilic acid	0.37	ND
Gentisic acid	0.11	0.47
Caffeic acid	ND	1.73
Sinapic acid	5.73	2.92
Trans-cinnamic acid	5.71	4.99
Chlorogenic acid	2.19	4.26
-Catechin gallate	1.27	1.26
Procianidin	0.62	0.96
Myricetin	1.03	1.51
Kaempferol	1.56	2.26
Rutin	0.41	0.75
Narigin	0.59	1.84

ND: not detected.

## Data Availability

Data are available upon request.

## Supplementary Materials

The following are available online, File 1: IAV LC/MS-MS Data, File 2: AAV LC/MS-MS Data.

## Sample Availability

Samples of the compounds are available from the authors.
